# Microfluidic Bioreactor Made of Cyclo-Olefin Polymer for Observing On-Chip Platelet Production

**DOI:** 10.3390/mi12101253

**Published:** 2021-10-15

**Authors:** Hiroki Kumon, Shinya Sakuma, Sou Nakamura, Hisataka Maruyama, Koji Eto, Fumihito Arai

**Affiliations:** 1 Department of Micro-Nano Mechanical Science and Engineering, Nagoya University, Nagoya 464-8603, Japan; hisataka@mech.nagoya-u.ac.jp; 2 Department of Mechanical Engineering, Kyushu University, Fukuoka 819-0395, Japan; sakuma@mech.kyushu-u.ac.jp; 3 Center for iPS Cell Research and Application, Kyoto University, Kyoto 606-8507, Japan; sou.nakamura@cira.kyoto-u.ac.jp (S.N.); kojieto@cira.kyoto-u.ac.jp (K.E.); 4 Department of Mechanical Engineering, The University of Tokyo, Tokyo 113-8656, Japan; arai-fumihito@g.ecc.u-tokyo.ac.jp

**Keywords:** bioreactor, microfluidic chip, three-dimensional microchannel, fabrication, platelet, cyclo-olefin polymer

## Abstract

We previously proposed a microfluidic bioreactor with glass–Si–glass layers to evaluate the effect of the fluid force on platelet (PLT) production and fabricated a three-dimensional (3D) microchannel by combining grayscale photolithography and deep reactive ion etching. However, a challenge remains in observing the detailed process of PLT production owing to the low visibility of the microfluidic bioreactor. In this paper, we present a transparent microfluidic bioreactor made of cyclo-olefin polymer (COP) with which to observe the process of platelet-like particle (PLP) production under a bright-field, which allows us to obtain image data at a high sampling rate. We succeeded in fabricating the COP microfluidic bioreactor with a 3D microchannel. We investigated the bonding strength of COP-COP layers and confirmed the effectiveness of the microfluidic bioreactor. Results of on-chip PLP production using immortalized megakaryocyte cell lines (imMKCLs) derived from human-induced pluripotent stem cells show that the average total number of produced PLPs per imMKCL was 17.6 PLPs/imMKCL, which is comparable to that of our previous glass–Si–glass microfluidic bioreactor (17.4 PLPs/imMKCL). We succeeded in observing PLP production under a bright-field using the presented microfluidic bioreactor and confirmed that PLP fragmented in a narrow area of proplatelet-like protrusions.

## 1. Introduction

Platelet (PLT) products for blood transfusion are made from donated blood. However, the supply of PLT products is expected to be short owing to a decreasing number of blood donors [1,2,3]. Therefore, artificial non-donated PLT products are crucial to meeting increasing demand. Studies on the in vitro production of PLTs were conducted using microfluidic chips to elucidate the mechanism of PLT production [4,5,6]. Microfluidic bioreactors are expected to be used as small test benches for the in vitro evaluation of PLT production. These microfluidic bioreactors are designed to have microchannels with microstructures for trapping the injected megakaryocytes (MKs). Microfluidic bioreactors in the form of microfluidic chips are expected to be used in the evaluation of detailed PLT production processes through microscopic observation with low sample consumption.

**Figure 1:**
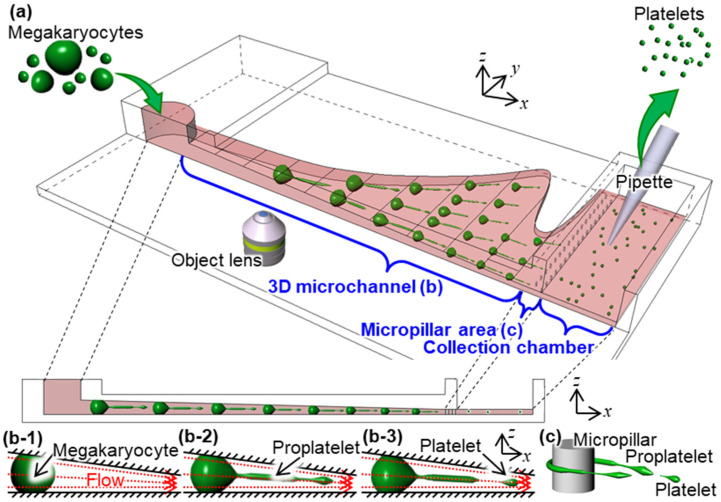
On-chip PLT production using a 3D microchannel. (**a**) Conceptual image of the microfluidic bioreactor, (**b**) Procedures of PLT production of (**b-1**) The trapping of an MK, (**b-2**) The elongation of an MK to a PPLT, and (**b-3**) The fragmentation of a PPLT to a PLT, and (**c**) Trapping of a released PPLT.

We previously proposed a microfluidic bioreactor with a three-dimensional (3D) microchannel for PLT production by utilizing a microfluidic bioreactor consisting of a three-layer glass–Si–glass structure, as shown in Figure 1 [7]. Glass–Si–glass microfluidic bioreactors have high rigidity and can easily be fabricated with a complex structure. We achieved a high-resolution 3D microchannel using the combination of grayscale photolithography and deep reactive ion etching (D-RIE) in previous our paper [7]. We succeeded in evaluating the effect of the fluid force on the production of platelet-like particles (PLPs) using the microchannel with a constant flow rate. While production efficiency has been the most instrumental point in in vitro culture devices, real-time visualization of thrombopoiesis under physical stimulation is also important for investigating the morphology of PLT production. We conducted the time-lapse monitoring of PLP production in the microfluidic bioreactor using immortalized megakaryocyte cell lines (imMKCLs) expressing the green fluorescent protein. We confirmed that the trapped imMKCLs elongated to proplatelet-like protrusions (PPLPs) and fragmented into PLPs. However, there is a challenge in observing the PLP production in detail. Since we could not observe the PLT production with transparent light because of the Si layer, a fluorescence observation was conducted for monitoring. An exposure time of 5 s was required to clearly observe imMKCLs with fluorescence. This exposure time depends on the excitation and fluorescence intensity. Ultimately, it was difficult to confirm the fragmented position in detail, because the morphology of imMKCLs changed appreciably during a period of 5 s at a flow velocity of 1 to 10 mm/s. Therefore, it was difficult to observe the detailed shape at the site of fragmentation of PLTs with a high sampling rate through fluorescence observation. Moreover, thin-string-shaped proplatelets (PPLT) could not be observed because of their low fluorescence intensity. Since real-time visualization of thrombopoiesis under physical stimulation is not well established and optimized yet, therefore, dynamic morphology of PLT production is not well studied. Under these circumstances, a transparent microfluidic bioreactor is desirable for observing PLT production. Polydimethylsiloxane (PDMS), which is a silicone elastomer, is frequently used as a material for transparent microfluidic bioreactors. PDMS is easy to use, inexpensive, and suitable for mass production [8]. However, Young’s modulus of PDMS is approximately 0.1 to 10 MPa [9], and the low rigidity of PDMS is not suitable for on-chip PLT production because an applied pressure of 10 to 200 kPa deforms the microchannel.

The purpose of this paper is the observation of the detailed shape at the fragmentation of PLTs under a bright-field with a high sampling rate. Owing to the high pressure applied, we propose using a transparent microfluidic bioreactor made of cyclo-olefin polymer (COP). First, we investigated the bonding strength of COP-COP layers of the microfluidic bioreactor in terms of whether these layers peel off when the pressure of 200 kPa was applied to the microchannel. Then, considering the measured tensile adhesive strength, we designed a 3D microchannel in a finite element method (FEM) analysis. We observed the process of PLP production and evaluated the number of produced PLPs to compare the PLP production of glass–Si–glass and COP microfluidic bioreactors. We succeeded in observing the PLP production under a bright-field using the presented microfluidic bioreactor and observed that the PLP fragmented in a narrow area of PPLP. The results of on-chip PLP production using imMKCLs derived from human-induced pluripotent stem cells (hiPSCs) show that the average total number of produced PLPs per imMKCL was 17.6 PLPs/imMKCL, which was comparable to that for the previous glass–Si–glass microfluidic bioreactor (17.4 PLPs/imMKCL). From these results, we confirmed that the proposed transparent and rigid microfluidic bioreactor can be applied as a test bench for evaluating on-chip PLP production.

## 2. Design of the Microfluidic Bioreactor

### 2.1. Bonding of COP-COP Layers

Typical transparent materials used for microfluidic chips are polymers represented by PDMS and glass. Microfluidic chips made of PDMS are easy to process and suitable for mass production but do not have high rigidity. The maximum width of the microchannel is as large as 10 mm, and the maximum pressure that is applied to the microchannel is as high as 200 kPa in our design [7]. Therefore, a microfluidic chip made of PDMS is not suitable for on-chip PLT production owing to the large deformation of the microchannel. On the other hand, microfluidic chips made of glass have high rigidity, but it is not easy to fabricate complex structures with a 3D microchannel with micropillars from glass. Microfluidic chips are often made disposable in biochemical fields because a large number of experiments need to be performed, yet a microfluidic chip made of glass is not suitable for mass production. Under these circumstances, there has recently been growing interest in fabricating microfluidic devices made of thermoplastic materials, such as COP [10,11,12]. The advantages of COP material are transparency, biocompatibility, productivity, and chemical durability against organic solvents. COP with Young’s modulus of 2.1 GPa has better mechanical properties than PDMS and is easier to manufacture than Si and glass. Furthermore, COP can be fabricated using a variety of techniques. Therefore, we choose a COP of thermoplastic resin with the consideration of transparency, processability, biocompatibility, and rigidity. A major issue with COP chip fabrication is the bonding of COP-COP layers. Thermal bonding was used as a direct bonding method where no additional materials are utilized to the interface [13,14,15,16]. In this paper, to bond at a temperature lower than the glass transition temperature of the substrate material, a pretreatment was applied using oxygen plasma before bonding. This direct bonding method enables COP bonding with negligible deformation of the microchannel structure for on-chip PLT production.

### 2.2. Tensile Test for Measuring Bonding Strength

**Figure 2:**
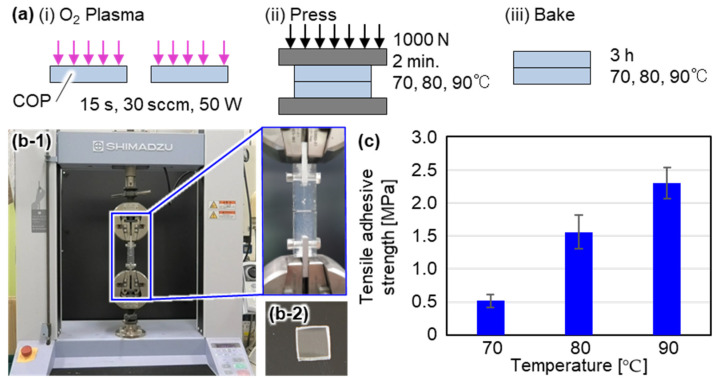
Bonding of COP-COP layers. (**a**) COP-COP bonding procedure, (**b-1**) Photographs of the tensile test device, (**b-2**) Photographs of the COP plate used in the evaluation of the tensile adhesive strength, and (**c**) Evaluation of the tensile adhesive strength.

First, we evaluated the bonding strength of COP-COP layers in designing the microfluidic bioreactor in terms of whether these layers peeled off when the pressure of 200 kPa was applied to the microchannel. In this paper, we used COP with a glass transition temperature of 100 °C (ZEONOR 1060R, Zeon Co., Ltd., Tokyo, Japan). Figure 2a shows the bonding procedure of COP-COP layers. The two COP layers were bonded by hydrophilizing the surface using oxygen plasma. The plasma exposure time, flow rate of oxygen gas and reactive ion etching (RIE) power of the plasma were respectively set at 15 s, 30 sccm, and 50 W. To increase the bonding strength, a 1000 N force was applied for 2 min while heating the bonded COP-COP layers. Then, we baked the COP for 3 h. Note that the baking temperature was set at 70 °C, 80 °C, and 90 °C which are lower than a glass-transition temperature of COP of 100 °C. We evaluated the adhesive strength using a tensile test device, as shown in Figure 2b-1. COP plates with dimensions of 10 mm × 10 mm × 1 mm were used in measuring the bonding strength, as shown in Figure 2b-2. The bonded sample was glued with a fix jig for chucking on a tensile test system using xylene, and the sample was fixed and pulled perpendicularly by a tensile test system. The tensile adhesive strength P was evaluated using Equation (1).

(1) $$P=\frac{F_{max}}{A_{bond}}$$

where $F_{max}$ shows the maximum force at the moment of peeling off and $A_{bond}$ shows the bonding area. Figure 2c shows the relationship between the baking temperature and the tensile adhesive strength. From Figure 2c, we can see a tendency that the tensile adhesive strength increases with the baking temperature. Therefore, we set the bonding temperature at 90 °C, which indicated that a tensile adhesive strength of 2.3 MPa should be considered in designing the microfluidic bioreactor.

### 2.3. Design of the Microchannel

**Figure 3:**
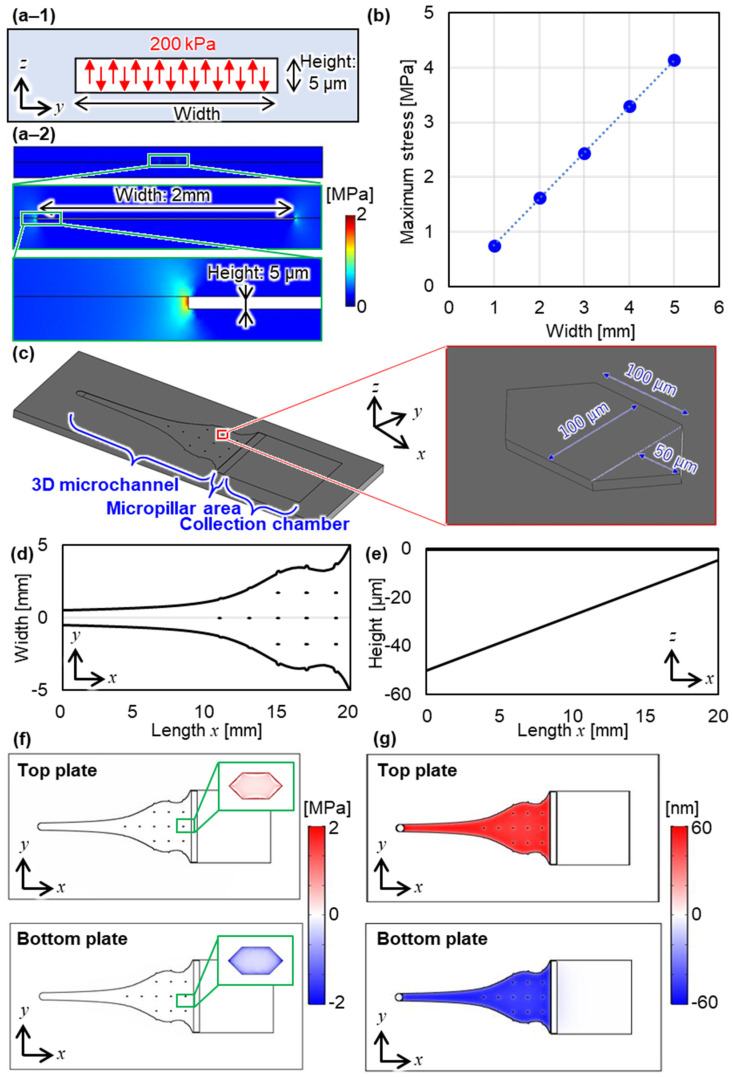
Design of the microchannel. (**a-1**) Model configuration and (**a-2**) Finite element method (FEM) image of the stress and (**b**) Analysis result of the relationship between the maximum stress and width of the microchannel. Considerations in the design of the microchannel. (**c**) Model configuration of the microfluidic bioreactor, (**d**) Width of the designed microchannel, (**e**) Height of the designed microchannel, (**f**) FEM image of the stress, and (**g**) FEM image of the deformation of the designed microfluidic bioreactor.

We designed the 3D microchannel using the basic evaluation results of the bonding strength. We designed the microfluidic bioreactor with two considerations of the bonding strength and the deformation of the microfluidic channel. Since the stress acting on the bonding surface increases with the increase in the area of the microchannel and applied pressure to the microchannel, we evaluated the relationship of the bonding strength of the COP-COP layers and the stress acting on the bonding surface by using FEM analysis (COMSOL Multiphysics v5.2, COMSOL AB, Stockholm, Sweden). Figure 3a-1,a-2 shows the two-dimensional model with the cross-section of bonding COP including microchannel in the FEM analysis, and the typical result of the stress when the width of the microchannel is 2 mm, respectively. Note that we used structural analysis and accordingly set that 200 kPa of the actual pressure was applied to the microchannel. The height of the microchannel was set as 5 µm. Figure 3b shows the relationship between the width of the microchannel and stress when pressures applied to the microchannel were set as 200 kPa in this simulation. The results show that the stress applied to the bonding surface increases with the width of the microchannel. Therefore, we added the micropillars to decrease the maximum stress acting on the bonding surface into the basic design of the microchannel presented in previous our paper [7]. By adding the micropillars with the pitch of 2 mm, we keep the maximum stress under 2.3 MPa which is the adhesive strength of COP when the bonding temperature was 90 °C, as shown in Figure 3c–e. Note that the width of the microchannel was designed to make the flow conditions constant [7]. Using the designed microchannel, we analyzed the stress on the bonding surface and the deformation of the microfluidic channel, as shown in Figure 3f,g. The stress and deformation were calculated by structure analysis. The geometric model of the microfluidic bioreactor was the same concept as that of the actual microfluidic bioreactor except for the shape of the micropillar area near the outlet. The micropillar area was replaced with a rectangular parallelepiped when analyzing the entire microfluidic bioreactor to reduce the computational cost in the analysis. The boundary condition was set as 200 kPa which is the same as the condition with actual pressure. Figure 3f shows the stress applied to the bonding surface in the FEM analysis. Note that after analyzing with a 3D model in which the top and bottom plates are assembled, the results are shown separately for each plate. Figure 3g shows the deformation of the microchannel in the FEM analysis. From these results, the maximum stress on the bonding surface was 1.8 MPa, and the maximum deformation was 54 nm. From these results, we estimated that the bonded layer does not peel off under the applied pressure because the maximum stress is less than the bonding strength.

## 3. Fabrication of the Microfluidic Bioreactor

### 3.1. Fabrication Process of the Microfluidic Bioreactor

**Figure 4:**
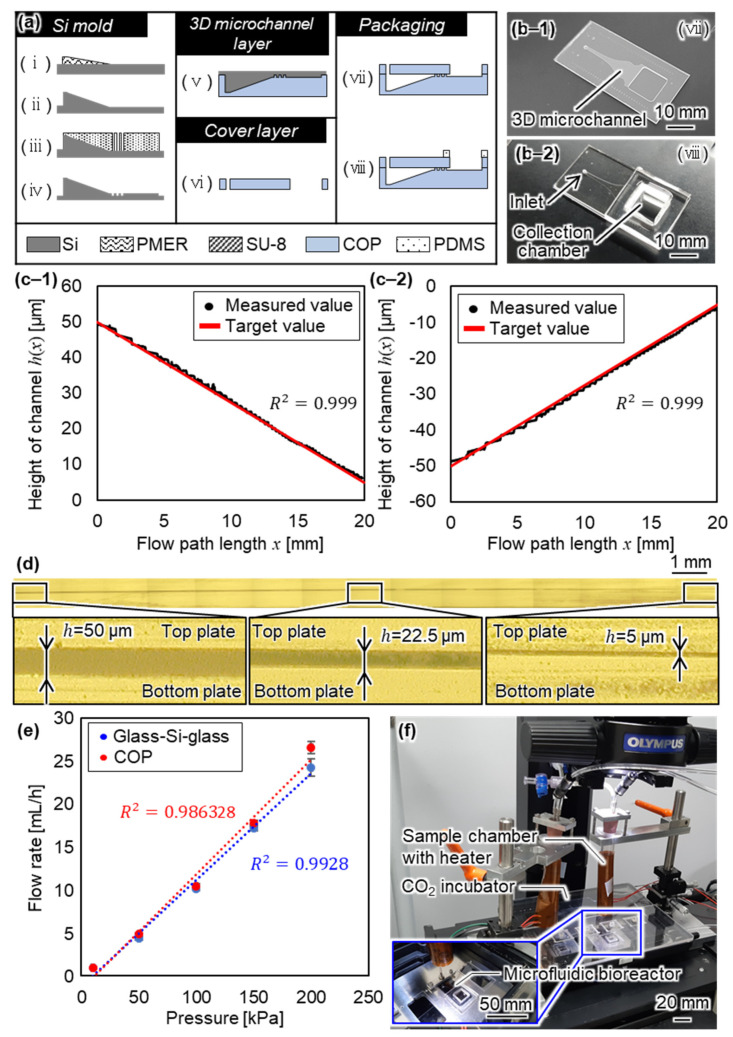
Fabrication of the COP microfluidic bioreactor. (**a**) Process flow of the fabrication of the microfluidic bioreactor, (**b-1**) Photograph after the bonding of COP-COP layers, (**b-2**) Photograph after bonding of the COP-PDMS layers, (**c**) Height evaluation of (**c-1**) The fabricated Si mold and (**c-2**) The fabricated microchannel of the COP, (**d**) Merged image of the cross-section of the 3D microchannel after bonding, (**e**) Evaluation result of the flow rate, and (**f**) Photograph of the constructed system.

The proposed microfluidic bioreactor consists of two COP layers as a cover, a 3D microchannel, and a collection chamber of PDMS. As our 3D microchannel has a sloped structure that traps imMKCLs of various sizes, we used a gray-scale lithography technique based on the resolution and size of the exposure area. We fabricated a Si mold of the 3D microchannel using the method based on a D-RIE. We transferred the 3D microchannel to a COP substrate using imprint techniques. The fabrication process flow and the fabricated bioreactor are shown in Figure 4a,b-1,b-2 respectively. The fabrication process is summarized as follows.

- For the 3D microchannel layer of the Si mold, PMER positive-type photoresist (Tokyo Ohka Co., Ltd., Tokyo, Japan) was patterned on the surface of the Si substrate using a gray-scale lithography technique. In this process, we directly wrote a pattern designed with an 8-bit gray-scale through laser scanning by changing the intensity.
- The Si substrate was etched using D-RIE. The 3D surface of the photoresist was transferred to the Si substrate according to the selective ratio.
- An SU-8 3005 negative-type photoresist (Microchem Co., Ltd., Tokyo, Japan) was patterned on the Si substrate. The SU-8 layer was used as an etching mask in the D-RIE to fabricate the pillar array of the microchannel.
- The pillar array of the microchannel was fabricated using D-RIE, and the remaining photoresist was then removed in the cleaning process.
- The structure of the microchannel was transferred from the Si mold to the COP of the thermoplastic resin using nanoimprint technology. The imprint temperature and force were respectively set at 140 °C and 1000 N.
- The inlet and collection chamber of the cover layer were fabricated by machining. We annealed the COP substrate after machining to remove burring. The temperature and force of annealing were set at 90 °C and 1000 N.
- The two fabricated layers were bonded after the surface was hydrophilized using oxygen plasma. The time, flow rate of oxygen gas and RIE power of plasma were respectively set at 15 s, 30 sccm, and 50 W. A force was applied while heating the bonded COP to increase the bonding strength. The temperature, force, and time were respectively set at 90 °C, 1000 N, and 2 min. Then, we baked the microfluidic bioreactor at 90 °C for 3 h.

### 3.2. Evaluation of the Fabricated Microfluidic Bioreactor

We evaluated the fabricated microfluidic bioreactor to investigate the characteristics of the microchannel. First, we measured the height of the 3D microchannel of the imprinted COP. Figure 4c-1,c-2 show the heights of the Si mold and imprinted COP, respectively. Figure 4d shows a merged image of the cross-section of the 3D microchannel after bonding. We confirmed that the structure of the 3D microchannel was transferred from the Si mold to the COP. As a result, we obtained the coefficient of determination R^2^ and the standard deviation of relative error against the design value of 0.999 and 0.55 µm (Si mold) and 0.999 and 0.72 µm (imprinted COP), respectively. Second, we measured the width of the 3D microchannel of the imprinted COP at the position of 19 mm from the inlet. Typical widths of the microchannel design, microchannel of the Si mold, and fabricated microchannel of the COP were 7.50, 7.46, and 7.43 mm, respectively. From these results, we succeeded in fabricating a COP microfluidic bioreactor with a 3D microchannel. Finally, we evaluated the flow rate into the microchannel versus the applied pressure because the material of the microfluidic bioreactor was different from the glass–Si–glass of our previously fabricated microfluidic bioreactor. Figure 4e shows the relationship between the flow rate and pressure for the glass–Si–glass and COP microfluidic bioreactors. The flow rate of the COP microfluidic bioreactor was similar to that of the glass–Si–glass microfluidic bioreactor. It is considered that the COP microfluidic bioreactor has the same pressure drop as the glass–Si–glass microfluidic bioreactor, and we can therefore investigate the effect of the fluid force on PLP production using the same procedure as before. We confirmed that pressure of 200 kPa can be applied to the microfluidic bioreactor without the COP layers peeling off and without leakage at the bonding surface.

### 3.3. System Configuration of the Microfluidic Bioreactor

Figure 4f shows the constructed on-chip PLP production system. The microfluidic bioreactor was set on an inverted microscope (IX73, Olympus Co., Ltd., Tokyo, Japan). The experimental procedure consisted of a loading state, producing state, and flushing state. In the loading state, an imMKCL suspension was injected into the microchannel by applying a pressure of 10 kPa for 15 min, as controlled by a pressure pump (Fluigent Co., Ltd., Paris, France). In the producing state, the medium was injected by applying pressure of 10 kPa to the chambers considering a preferable condition of PLP production obtained in our previous research [7]. Under this condition, the maximum shear rate was obtained in FEM analysis as 3.7 × 10^3^/s for an applied pressure of 10 kPa. Note that shear rate was calculated by computational fluid dynamics. We calculated the shear rate using the geometric model of the micropillar area because the shear rate becomes largest in the micropillar area near the outlet. We determined the measured flow rate when the pressure of 10 kPa was applied to the microchannel as the boundary condition. In the producing state, PLPs were produced by applying arbitrary pressure, where the time period was set at 3 h for the evaluation of the number of produced PLPs. In the flushing state, the medium was injected into the microchannel by applying pressure of 200 kPa for 15 min to collect the produced PLPs. The temperature of the sample chambers was maintained at 37 °C using flexible heaters (KH-303, Omega Engineering Inc., Tokyo, Japan). The temperature of the microfluidic bioreactor was maintained at 37 °C in a humidified 5% CO_2_ stage-top incubator (INUG2H-WSKM, TOKAI HIT Co., Ltd., Fuji, Japan).

## 4. Experiment on PLT Production

### 4.1. Monitoring of PLP Production

**Figure 5:**
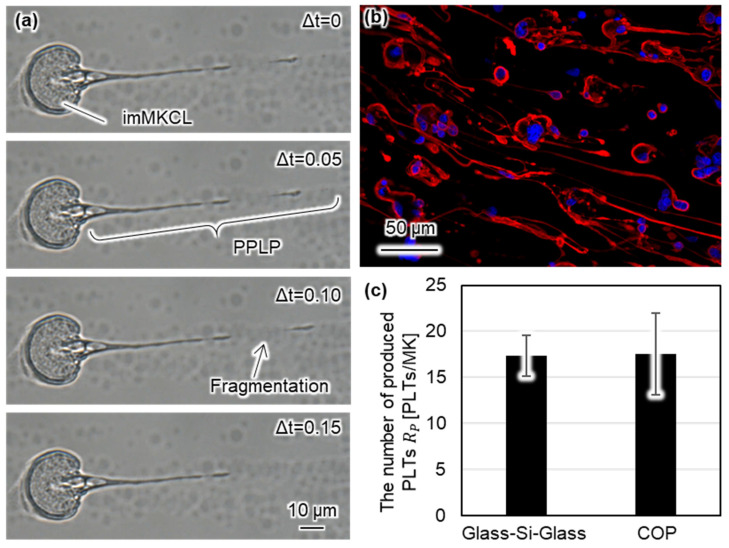
Experimental results of PLP production. (**a**) Bright-field image of PLP production, (**b**) Fluorescence image of PLP production, and (**c**) Evaluation of the number of produced PLPs.

Using the constructed system, we conducted monitoring of PLP production in the microfluidic bioreactor. First, we observed the PLT production under a bright field, as shown in Figure 5a. The supplementary movie Video S1 shows the detailed PLP production behavior. We recorded the PLT production at a frame rate of 20 fps using a CCD camera. We confirmed that the PPLP was fragmented by the fluid force in a narrow area. We succeeded in observing PLT production under a bright-field with a high sampling rate using the COP microfluidic bioreactor. Moreover, we conducted a fluorescence observation of PLP production after 1 h of the producing state. Figure 5b shows typical results for PLP production, where blue and red colors show the nucleus and β1-tubulin. The trapped imMKCLs were elongated to PPLPs. Thus, we succeeded in the fluorescence observation of PLP production using the COP microfluidic bioreactor.

### 4.2. Number of Produced PLPs

We evaluated the number of produced PLPs for the fabricated COP microfluidic bioreactor and the previous glass–Si–glass microfluidic bioreactor. This experiment used imMKCLs derived from hiPSCs and cultured in flasks for 5 days [1]. In the experiments, we used imMKCL suspensions with a concentration of 5.0 × 10^4^ cells/mL, and the total volume of the imMKCL suspension was 50 μL. The PLPs were counted using a commercial flow cytometer (FACSVerse, Franklin Lakes, NJ, USA) with the indexes of anti-hCD41-APC (Biolegend Inc., San Diego, CA, USA) and anti-hCD42b-PE (Biolegend Inc., San Diego, CA, USA). The total number of produced PLPs per one imMKCL $R_{total}$ was evaluated using Equation (2).

(2) $$R_{total}=\frac{\sum_{t=1}^{3}N_{produce}\left\{t\right\}+N_{flush}}{N}$$

where $N_{produce}\left\{t\right\}$ shows the number of PLPs collected at time t, $N_{flush}$ shows the number of PLPs collected in the flushing state, and N shows the total number of injected MKs. Since the imMKCL suspension contained PLPs before injection, the number of PLPs collected in the loading state was not added to the total number of collected PLPs. Three experiments were conducted using the COP and glass–Si–glass microfluidic bioreactors. Figure 5c shows the total number of produced PLPs per one imMKCL using the COP and glass–Si–glass microfluidic bioreactors. The average total number of produced PLPs per imMKCL were 17.4 and 17.6 PLPs/imMKCL when using the glass–Si–glass and COP microfluidic bioreactors, respectively. We analyzed the diameter of collected PLPs from the collection chamber with a CCD camera. From this result, the average and standard deviation of the diameter of the produced PLPs in the collection chamber of the glass–Si–glass and COP microfluidic bioreactors were 4.3 ± 1.2 μm and 4.1 ± 1.2 μm, respectively. From these results, we confirmed that the PLP production ability of the COP microfluidic bioreactor was comparable to that of the previous glass–Si–glass microfluidic bioreactor. Through evaluations of the number of produced PLPs, we confirmed that the COP microfluidic bioreactor can be applied as a test bench to evaluate on-chip PLP production.

## 5. Discussion

### 5.1. Versatility of the COP Microfluidic Bioreactor

We discuss the productivity of the COP microfluidic bioreactor. The fabrication of the COP microfluidic bioreactor is easy and productive. Microfluidic bioreactors are often made disposable in the biochemical fields. COP is easier to process and allows the manufacture of complex structures in comparison with traditional materials, such as Si and glass. COP can be processed using a variety of techniques, including microinjection molding, hot embossing, casting, reactive ion etching, and mechanical and laser micromachining, making it easier to standardize and handle and amenable to industrial manufacturing. In this paper, we fabricated a Si mold of a 3D microchannel by combining grayscale photolithography and D-RIE. Then, we transferred the 3D microchannel to a COP substrate using imprint techniques. The imprinted COP substrate was bonded COP-COP layers using oxygen plasma. The imprint method is suitable for the mass production of the microfluidic bioreactor. It is possible to manufacture the transparent and rigid microfluidic bioreactor in a highly productive manner while maintaining the functions of the previous glass–Si–glass microfluidic bioreactor.

### 5.2. Visibility of the COP Microfluidic Bioreactor

While production efficiency has been the most instrumental point in in vitro culture devices, real-time visualization of thrombopoiesis under physical stimulation is not well established and optimized yet. The observation of PLP production using the previous glass–Si–glass microfluidic bioreactor was limited to fluorescence observations. The frame rate of the fluorescence observation required to clearly observe imMKCLs was 0.2 fps, and we, therefore, could not observe the detailed shape at the site of fragmentation of PLPs. Moreover, the thin-string-shaped PPLTs could not be observed because of their low fluorescence intensity. In this paper, we presented a transparent COP microfluidic bioreactor for the observation of PLP production under a bright field, which allows us to obtain image data at a high sampling rate. As a result, we recorded PLT production at a frame rate of 20 fps under a bright-field using the presented transparent microfluidic bioreactor. This frame rate is 100 times shorter than that for the previous microfluidic bioreactor. We confirmed that the presented transparent microfluidic bioreactor can observe the dynamic morphology of PLT production at a higher sampling rate than the previous microfluidic bioreactor. By observing under a bright field, we could record the morphology immediately before and after the fragmentation of PPLP and confirmed that PPLP fragmented in a narrow area. Thus, the results confirm that we can observe PLT production under a bright-field with a high sampling rate using the COP microfluidic bioreactor. We can investigate the effects of fluid conditions and biological factors by measuring the length, elongation speed, and fragmentation location of PPLPs under a bright field. In the future, real-time visualization along with dynamic evaluation of extracellular and intracellular events should enable us to further develop in vitro PLT production system.

## 6. Conclusions

We presented a transparent COP microfluidic bioreactor that can be used to observe PLP production under a bright-field, allowing us to obtain image data at a high sampling rate. Considering the measured tensile adhesive strength, we designed a 3D microchannel for PLT production and succeeded in fabricating a COP microfluidic bioreactor with a 3D microchannel. We succeeded in observing the PLP production under a bright-field, where the sampling rate of recording was 100 times that of the microfluidic bioreactor presented in our previous paper. We confirmed that the PLP had fragmented in a narrow area of PLPs. Results of on-chip PLP production using imMKCLs derived from hiPSCs show that the average total number of produced PLPs per imMKCL was 17.6 PLPs/imMKCL, which was comparable to that for the previous glass–Si–glass microfluidic bioreactor (17.4 PLPs/imMKCL). The proposed microfluidic bioreactor can be applied as a test bench for evaluating on-chip PLP production. The proposed method will contribute not only to evaluating PLP production but also to observing PLP production in microscopic observations.

## Supplementary Materials

The following are available online at https://www.mdpi.com/article/10.3390/mi12101253/s1, Video S1: Bright-field movie of PLP production.
